# Histological Lesions, Cell Cycle Arrest, Apoptosis and T Cell Subsets Changes of Spleen in Chicken Fed Aflatoxin-contaminated Corn 

**DOI:** 10.3390/ijerph110808567

**Published:** 2014-08-20

**Authors:** Xi Peng, Keying Zhang, Shiping Bai, Xuemei Ding, Qiufeng Zeng, Jun Yang, Jing Fang, Kejie Chen

**Affiliations:** 1 College of Veterinary Medicine, Sichuan Agricultural University, Sichuan 625014, China; E-Mails: pengxi197313@163.com (X.P.); fangjing4109@163.com (J.F.); ckj930@126.com (K.C.); 2 Key Laboratory for Animal Disease-Resistance Nutrition of China Ministry of Education, Institute of Animal Nutrition, Sichuan Agricultural University, Sichuan 625014, China; E-Mails: shipingbai@aliyun.com (S.B.); dingxuemei0306@163.com (X.D.); zqf@sicau.edu.com (Q.Z.); yangjun0612@163.com (J.Y.)

**Keywords:** aflatoxin, broiler, pathological lesion, apoptosis, cell cycle

## Abstract

The purpose of this study was to evaluate the effects of corn naturally contaminated with aflatoxin B1 and aflatoxin B2 on pathological lesions, apoptosis, cell cycle phases and T lymphocyte subsets of spleen, and to provide an experimental basis for understanding the mechanism of aflatoxin-induced immunosuppression. A total of 900 COBB500 male broilers were randomly allocated into five groups with six replicates per group and 30 birds per replicate. The experiment lasted for 6 weeks and the five dietary treatments consisted of control, 25% contaminated corn, 50% contaminated corn, 75% contaminated corn and 100% contaminated corn groups. The histopathological spleen lesions from the contaminated corn groups was characterized as congestion of red pulp, increased necrotic cells and vacuoles in the splenic corpuscle and periarterial lymphatic sheath. The contaminated corn intake significantly increased relative weight of spleen, percentages of apoptotic splenocytes, induced cell cycle arrest of splenocytes, increased the percentages of CD3^+^CD8^+^ T cells and decreased the ratios of CD3^+^CD4^+^ to CD3^+^CD8^+^. The results suggest that AFB-induced immunosuppression maybe closely related to the lesions of spleen.

## 1. Introduction

Mycotoxins are secondary toxic fungal metabolites which cause important health problems in human and animals [1]. Aflatoxins are a type of mycotoxin which usually contaminate feed ingredients during storage. Among identified aflatoxins, aflatoxin B1 (AFB1) is the predominant form, which presents the highest hepatotoxic and carcinogenic effects [2] and is classified as a Group I carcinogen to humans by the International Agency for Research on Cancer [3]. The toxic and carcinogenic effects of AFB1 are closely linked with its biotransformation [4]. The active intermediate, AFB1-exo-8, 9-epoxide, can bind with DNA to form the predominant *trans*-8, 9-dihydro-8-(N7-guanyl)-9-hydroxy-AFB1 (AFB1-N7-Gua) adduct which causes DNA lesions [5]. Previous studies proved that aflatoxins had a range of negative effects on poultry health. Besides carcinogenic effects [6], acute or chronic aflatoxicosis in poultry birds results in poor performance [7], immunosuppression, and increased susceptibility to disease [6,8]. 

As the largest peripheral lymphoid tissue of the body, the spleen is of vital importance in the whole immune function [9]. Previous studies showed that AFB1 caused tissue damage of spleen in rats [10], induced bio-molecular oxidative damage and decreased cell proliferation in spleen mononuclear cells (SMC) [11,12], induced mutations of splenic lymphocytes from rats [13], and decreased the number of CD4^+^ and CD8^+^ T cells in mice [14].

Although lymphocytic depletion of spleen in broilers and ducklings exposed to AFB has been reported [15,16], there are no systemic studies on spleen damage in broilers exposed to AFB. AFB1 22 is commonly found along with AFB2 in corn in the southwest of China, so the objectives of this study were to evaluate the pathological lesion, apoptosis and T cell subsets of spleen in broilers fed on corn naturally contaminated with AFB1 and AFB2. The results could aid in understanding the underlying basis for the immunosuppression attributable to aflatoxin. 

## 2. Materials and Methods

### 2.1. Animals, Diets and Study Design

Nine hundred 1-day-old Cobb500 chickens were randomly assigned to five experimental groups with six replicates per group and 30 birds per replicate. The birds were housed in the cages for 42 days at the Animal Nutrition Institute of Sichuan Agricultural University in China. Chicks were provided the corresponding diets and water *ad libitum* throughout the 42 days of experimentation. Room lights were set on a 24-h continuous schedule, temperature was initially maintained at 31 °C and gradually lowered by 2 °C each week until 21 °C, and relative humidity were maintained between 65% and 67%. The animal experiment was conducted in accordance with guidelines approved by Animal Health and Care Committee of Sichuan Agricultural University.

The control animals were fed with the corn-soybean basal diet. Nutritional requirements of the diet were adequate according to National Research Council (1994) and Agricultural Trade Standardization of China (NY/T33-2004). The composition and nutrient levels of the diet were described previously [17]. The basal control diet was not contaminated with AFB1 and AFB2. The four treated groups were given diets in which the ratio of naturally contaminated corn as a substitute for normal corn was 25%, 50%, 75%, and 100%, respectively. By the method of high performance liquid chromatography (Agilent 1100, Forster City, CA, USA), the contents of mycotoxins, including AFB1, AFB2, AFG1, AFG2, T-2 toxin, deoxynivalenol (DON), zearalenone (ZEN), ochratoxin A (OTA), and fumonisin B1 (FB1) was detected as described previously [18]. The detection limits of above mycotoxins were 2 µg/kg for AFB1, 0.8 µg/kg for AFB2, 2.5 µg/kg for AFG1, 1.5 µg/kg for AFG2, 100 µg/kg for T-2 toxin, 300 µg/kg for DON, 100 µg/kg for ZEN, 30 µg/kg for OTA, and 200 µg/kg for FB1 [19]. 

The results showed that naturally contaminated corn used in the diet was mainly contaminated with AFB1 and AFB2. The AFB1 contents in diets were 16.3~82.4 µg/kg in the starter period and 34.3~134 µg/kg in the grower period (Table 1). The AFB2 concentrations in diets were 3.15~14.2 and 6.17~23.6 µg/kg in the starter and grower periods, respectively. The contents of AFB1 and AFB2 were different due to different storage time and contaminated degree of corn (Table 1). The contents of other mycotoxins (including AFG1, AFG2, DON, ZEA, OTA, T-2 toxin, and FB1) were below the limit of detection.

At 21 and 42 days of age during the experiment, birds were sacrificed; spleens were sampled for the pathological observation and the determination of the cell cycle, apoptosis, and T cell subsets by flow cytometry.

**Table 1:** Mycotoxins concentrations in diet and corn (air-dry basis μg/kg).

Diet ^1^	Control	25%	50%	75%	100%	Control Corn	Contaminated Corn
1~21d							
AFB_1_	ND ^2^	16.3	36.9	45.6	82.4	ND	149.6
AFB_2_	ND	3.15	6.38	7.86	14.2	ND	24.2
22~42d							
AFB_1_	ND	34.3	69.3	95.2	134	ND	229
AFB_2_	ND	6.17	12.1	17	23.6	ND	37.8

Notes: ^1^ Control = diet with control corn; 25% = diet with 25% naturally contaminated corn; 50% = diet with 50% naturally contaminated corn; 75% = diet with 75% naturally contaminated corn; 100% = diet with 100% naturally contaminated corn; ^2^ ND = not detectable.

### 2.2. Relative Weight of Spleen

At 21 and 42 days of age during the experiment, after the body weight was weighed, six birds in each group were euthanized and necropsied. The spleen was dissected from each chick, and weighed after dissecting connective tissue around the organ. Relative weight of organ was calculated through the following formula:

Relative weight = organ weight/body weight (mg/kg)
(1)

### 2.3. Pathological Observation

After weighing, spleens were fixed in 4% buffered formaldehyde and routinely processed in paraffin. Thin sections (5 μm) of each tissue were sliced from each block and mounted on glass. Slides were stained with hematoxylin and eosin Y. Histological slides were examined on an Olympus light microscope.

### 2.4. Cell Cycle Phase Detection

At 21 and 42 days of age during the experiment, six birds in each group were euthanized. The spleen was dissected from each chick and immediately minced with surgical scissors. The cell suspension was filtered through a 300-mesh nylon mesh, washed twice with 0.1 M (pH 7.4) cold phosphate buffered saline (PBS), and then resuspended cells in PBS at a concentration of 1 × 10^6^ cells/mL. The 1 mL suspension was transferred to a 5-mL culture tube and centrifuged at 200× g for 5 min. The supernatant was discarded, and 1 mL PI staining solution (5 μL/mL propidium iodide, 0.5% Triton X-100, 0.5% RNase, PBS) was added. The cells were gently vortexed and incubated for 20 min at room temperature (25 °C) in the dark. 2 mL PBS were added and centrifugal elutriation performed once. The supernatant was discarded. The cells were re-suspended in 0.5 mL PBS and the cell phases were analyzed by flow cytometry (FACSCalibur, BD, Franklin Lake, NJ, USA).

### 2.5. Apoptosis Detection

At 21 and 42 days of age during the experiment, six birds in each group were euthanized. Spleen was dissected from each chick and immediately minced with surgical scissors. The cell suspension was filtered through a 300-mesh nylon mesh, washed twice with cold PBS and then resuspended cells in 1× binding buffer (Cat. No. 51-66121E, BD Pharmingen, Santiago, CA, USA) at a concentration of 1 × 10^6^ cells/mL. One hundred microliters of the solution was transferred to a 5-mL culture tube, and then 5 μL of Annexin V-FITC (Cat. No. 51-65874X, BD Pharmingen, Santiago, CA, USA) and 5 μL of PI (Cat. No. 51-66211E, BD Pharmingen, Santiago, CA, USA) were added. The cells were gently vortexed and incubated for 15 min at room temperature (25 °C) in the dark. Four hundred microliters of 1× binding buffer was added to each tube and analyzed by flow cytometry (FACSCalibur) within 1 h.

### 2.6. Cell T Cell Subsets Detection by Flow Cytometry

The spleens of six birds in each group were taken to determine the percentages of CD3^+^, CD3^+^CD4^+^, CD3^+^CD8^+^ T cells by the flow cytometry method and calculate the CD4^+^/CD8^+^ ratio at 21 and 42 days of age during the experiment. Splenic single cell suspension was prepared by gently cutting each spleen and then filtering through nylon gauze. Splenic single cell suspension was centrifuged at 200× g for 5 min. The supernatant was discarded and lymphocytes were collected. The cell concentration was diluted to 1.0 × 10^6^ cells/mL with phosphate-buffered saline (PBS). 100 μL cell suspensions were transferred to another centrifuge tube, and respectively stained with 10 μL mouse anti-chicken CD3-SPRD (Cat. No. 8200-13, SouthernBiotech, Birmingham, AL, USA), mouse anti-chicken CD4-FITC (Cat. No. 8210-02, SouthernBiotech, Birmingham, AL, USA) and mouse anti-chicken CD8a-RPE (Cat. No. 8220-09, SouthernBiotech, Birmingham, AL, USA) for 15–20 min at room temperature, and then 2 mL PBS added and centrifugal elutriation performed once. The supernatant was discarded. The cells were resuspended in 0.5 mL PBS and determined using a BD FACS Calibur flow cytomyter.

## 3. Results

### 3.1. Relative Weight of Spleen

**Figure 1:**
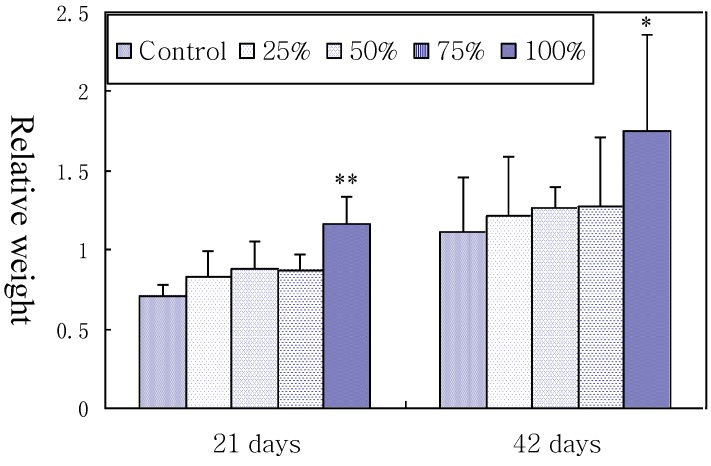
Effects of corn naturally contaminated with AFB1 and AFB2 on relative weight of broilers spleen. Data are presented with the means ± standard deviation (*n* = 6). When compared with the control group, * means *p* < 0.05. ** means *p* < 0.01.

As described in Figure 1, relative weight of spleen was significantly higher in 100% contaminated group than in control group at 21 and 42 days of age (*p* < 0.01 or *p* < 0.05), but no significant differences were observed among the control, 25%, 50% and 75% groups although there was an increase tendency. The results indicated that high level of simultaneous AFB1 and AFB2-contaminated diet intake increased splenic relative weight in broilers.

### 3.2. Pathological Lesions

**Figure 2:**
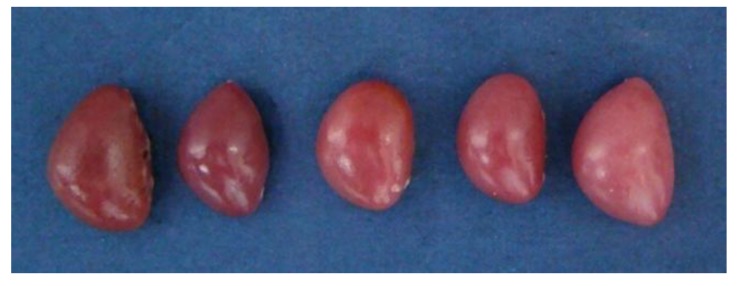
Gross changes in the size and colour of the spleen in the chickens at 21 days of age. From left to right: spleen in control group, 25%, 50%, 75% and 100% groups.

Macroscopically, the spleens were dark red in control group, but they were light red in the 50%, 75% and 100% groups (Figure 2), and spleen was increased in size, especially in the 100% group. The most obvious changes were observed at 21 days of age.

**Figure 3:**
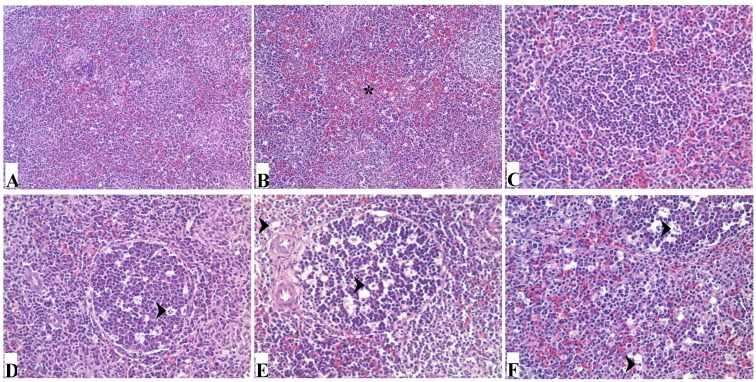
Photomicrographs of hematoxylin and eosin stained chicken spleen section. (**A**) shows the normal histological structure of control group; (**B**) shows that congestion of red pulp (*) is obvious in 100% group. At 21 days of age, when compared with those of control group (**C**), nuclear debris and vacuoles (arrows) are gradually increased in the spleen of chickens in 50%, 75% and 100% groups (**D**–**F**, respectively).

Histopathologically, there were no obvious lesions in the spleens of the 25% group when compared with those of control group (Figure 3A) at 21 and 42 days of age. Congestion of red pulp became obvious as the dietary mycotoxin level was increased (Figure 3B). Compared with the control group (Figure 3C), necrotic cells and vacuoles in the splenic corpuscle and periarterial lymphatic sheath of the spleen were gradually increased in number in the 50%, 75%, and 100% groups (Figure 3D–F) at 21 days of age. Histological lesions of spleen in mycotoxin contaminated corn groups were all alleviated at 42 days of age.

### 3.3. Cell Cycle Phase-Distribution of Splenocytes

**Figure 4:**
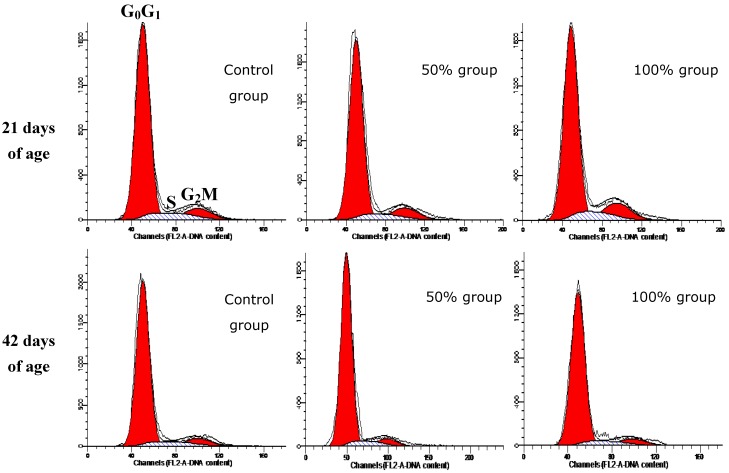
DNA histograms of splenocytes’ cell cycles. The percentage of G_2_M phase is higher in 50% and 100% group than in control group at 21 days of age. The percentage of S phase is lower in 50% and 100% group than in control group at 42 days of age.

At 21 days of age, the percentage of G_0_G_1_ splenocytes in the 100% group was lower than in the control group, and the proliferating index (PI) value in100% group was higher than control group (*p* < 0.05). Changes of the G_2_M phase were obvious; the percentages of G_2_M phase splenocytes were increased in a dose-dependent manner. However, at 42 days of age, when compared with control group, the percentages of splenocytes in G_0_G_1_ phase were obviously increased in the 75% and 100% groups (*p* < 0.01 or *p* < 0.05); the percentages of splenocytes in S phase and PI value were decreased (*p* < 0.01 or *p* < 0.05) in 75% and 100% groups; the percentage of G_2_M phase splenocytes was decreased in 100% group. The results showed that G_2_M and G_0_G_1_ phase blockage could be observed in chicken spleen at 21 and 42 days of age respectively. The results were shown in Table 2 and Figure 4.

**Table 2:** Effect of AFB-contaminated corn on cell cycle phase distribution of spleen in chickens.

Time	Group	G_0_G_1_ Phase (%)	G_2_M Phase (%)	S Phase (%)	PI
21 days	Control	82.04 ± 2.87	9.05 ± 1.51	8.91 ± 1.40	17.96 ± 2.87
	25%	80.95 ± 3.27	9.49 ± 1.56	9.55 ± 1.72	19.05 ± 3.27
	50%	79.60 ± 1.17	10.71 ± 0.74 *	9.69 ± 0.82	20.40 ± 1.17
	75%	79.32 ± 1.86	11.68 ± 1.28 **	9.00 ± 1.02	20.68 ± 1.86
	100%	78.37 ± 0.80 *	12.88 ± 0.50 **	8.74 ± 0.38	21.63 ± 0.80 *
42 days	Control	84.56 ± 0.67	7.69 ± 0.73	7.75 ± 0.41	15.44 ± 0.67
	25%	84.47 ± 2.12	7.50 ± 0.99	8.02 ± 1.14d	15.53 ± 2.12
	50%	85.52 ± 1.65	7.25 ± 0.91	7.23 ± 1.00	14.45 ± 1.65
	75%	86.80 ± 1.32 *	6.81 ± 0.64	6.39 ± 0.84 *	13.20 ± 1.32 *
	100%	87.42 ± 0.70 **	6.25 ± 0.22 **	6.33 ± 0.84 *	12.58 ± 0.70 **

Notes: Data are presented with the means ± standard deviation (*n* = 6). When compared with the control group, * means *p* < 0.05, ** means *p* < 0.01.

### 3.4. Annexin V-FITC Staining Assay by Flow Cytometry

**Figure 5:**
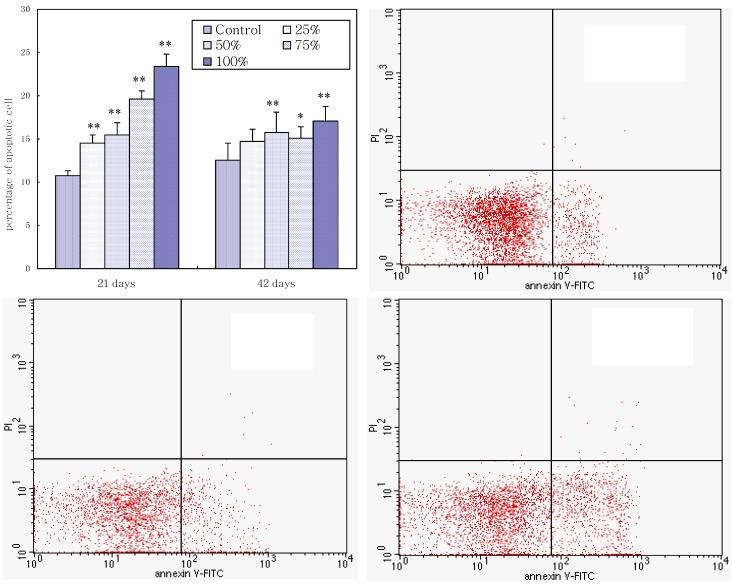
The upper left histogram shows the effects of corn naturally contaminated with AFB_1_ and AFB_2_ on apoptosis of splenocytes. Data are presented with the means ± standard deviation (*n* = 6). When compared with the control group, * means *p* < 0.05. ** means *p* < 0.01. Quadrantal diagram showed that a minor percentage of cells in control group are undergoing apoptosis and increased percentage of cells in 50% and 100% groups is undergoing apoptosis.

As shown in Figure 5, the percentages of apoptotic splenocytes increased as the dietary aflatoxin level increased. The percentages of apoptotic cells in the 25%, 50%, 75%, and 100% groups were significantly higher (*p* < 0.01) than those in the control group, and the percentages of apoptotic cells in the 75% and 100% groups were significantly higher (*p* < 0.01) than those in the 25% and 50% groups at 21 days of age. At 42 days of age, compared with the control group, the percentages of apoptotic splenocytes in the 50%, 75%, and 100% groups were increased (*p* < 0.01 or *p* < 0.05). A quadrantal diagram analyzed by flow cytometer showed that more splenocytes in AFB-contaminated groups were undergoing apoptosis (Figure 5).

### 3.5. Splenic T Cell Subsets

At 21 days of age, the percentages of CD3^+^CD8^+^ T cells in the 75% and 100% groups were much higher (*p* < 0.05) and the ratios of CD3^+^CD4^+^ T cells to CD3^+^CD8^+^ T cells were much lower (*p* < 0.05 or *p* < 0.01) than those in the control group. At 42 days of age, compared with the control group, the percentage of CD3^+^CD8^+^ T cells in the 100% groups was significant increased (*p* < 0.05), and the ratios of CD3^+^CD4^+^ to CD3^+^CD8^+^ were significant decreased (*p* < 0.05) (Table 3).

**Table 3:** Changes of splenic T-cell subsets (%).

Time	Items	CD3^+^	CD3^+^CD4^+^	CD3^+^CD8^+^	CD3^+^CD4^+^/CD3^+^CD8^+^
21 days	Control	64.73 ± 3.05	34.67 ± 2.08	27.47 ± 2.19	1.27 ± 0.08
	25%	65.38 ± 4.85	33.02 ± 6.06	32.34 ± 7.39	1.06 ± 0.30
	50%	64.13 ± 5.85	33.98 ± 4.84	32.17 ± 5.91	1.09 ± 0.30
	75%	62.26 ± 5.11	35.39 ± 5.68	36.04 ± 5.49 *	0.98 ± 0.07 *
	100%	59.57 ± 4.77	30.19 ± 1.96	34.34 ± 2.09 *	0.88 ± 0.08 **
42 days	Control	77.00 ± 5.26	31.83 ± 5.32	34.94 ± 3.48	0.91 ± 0.07
	25%	74.95 ± 4.83	30.11 ± 5.47	34.94 ± 6.59	0.89 ± 0.25
	50%	77.34 ± 2.94	29.34 ± 6.67	37.20 ± 2.92	0.79 ± 0.19
	75%	75.20 ± 3.81	31.37 ± 3.92	36.59 ± 2.44	0.86 ± 0.14
	100%	79.06 ± 5.13	26.65 ± 4.08	43.72 ± 5.91 **	0.62 ± 0.12 *

Notes: Data are presented with the means ± standard deviation (*n* = 6). When compared with the control group, * means *p* < 0.05, ** means *p* < 0.01.

## 4. Discussion

The spleen is known as the principal pheripheral immune organ. In the present study, the relative weight of spleen was used to judge the extent of spleen lesions. The relative weight of the spleen in birds fed with corn naturally contaminated with AFB_1_ and AFB_2_ increased, which was similar to the results from other researchers [20,21,22]. According to histopathological observation, the relative weight increase seems to be due to the congestion of red pulp in the spleen. As known, splenic nodules and the periarterial lymphatic sheath are where B lymphocytes and T lymphocyte gather and mature, respectively [23]. In the present study, histopathological results showed that necrotic cells in the splenic corpuscle and periarterial lymphatic of the spleen were gradually increased in number in the 50%, 75%, and 100% groups, which indicated that excessive necrosis of B cells and T cells would finally impair the immune function of the spleen. 

In the present study, the percentages of splenocytes in G_2_M phase increased in a dose-dependent manner at 21 days of age. Previous studies showed that AFB-induced cell cycle arrest occurred at different phases depending on the cell type. Thomas *et al*. reported that aflatoxin B1 treatment can lead to an accumulation of chicken thymocytes in G_2_M phase* in vitro* [24]; but others researchers found that AFB_1_ significantly increased the S-phase cell population in murine macrophases [25] and human bronchial epithelial cells [26]* in vitro*. The results of the present study suggested that more than 36.9 mg/kg AFB_1_ combined with 6.38 mg/kg AFB_2_ could induce G_2_M phase and G_0_G_1_ phase arrest in chicken splenocytes at 21 and 42 days of age, respectively, which could lead to cell cycle blockage, inhibit the proliferation of splenocytes, drive cells into apoptosis and accordingly decrease the numbers of functional immunocyte in the spleen. Why were the splenocytes arrested in different cell cycle phase at different periods? The mechanisms of cell cycle arrest induced by dietary AFB_1_ and AFB_2_ should be further evaluated in the future study.

Apoptosis is a mode of programmed cell death, which allows elimination of unnecessary cells to maintain tissue homeostasis [27]. However, it has been also proven that defective apoptotic processes are implicated in an extensive variety of diseases [28], and excessive apoptosis are actively involved in immunosuppression in various circumstances [29]. In our study, increased apoptotic splenocytes were observed in the AFB-contaminated groups by flow cytometry, and this result was in agreement with the histological observations, which showed that increased nuclear debris was present in the spleens of chicken exposed to AFB contaminated diets. Previous report showed that AFB_1_ was able to induce apoptosis in hepatocytes, bone marrow cells and lung cells, or human bronchial epithelial cells [30,31,32]. These results partly revealed the mechanisms of AFB-induced immunosuppression. The up-regulation of apoptotic splenocytes induced by AFB_1_ and AFB_2_ are associated with the following factors: (a) oxidative stress of splenocytes induced by aflatoxins [33]; (b) increase of the Bax/Bcl-2 ratio, caspase-3, and caspase-9 caused by AFB_1_ led to cellular apoptosis via a process that involves mitochondrial damage [34,35,36].

T lymphocytes are responsible for cell-mediated immunity and are further classified according to their expression of cell surface proteins. CD3 molecular is the surface marker of mature T cells, most CD4^+^ T cells are helper T cells, and CD8^+^ T cells are cytotoxic T cells [37]. Previous studies showed that the percentage of CD3**^+^** peripheral blood T cells was decreased [38], and the percentages of CD4^+^ T-cells and CD8^+^ T-cells of splenic lymphocytes were decreased in mice exposed to AFB_1_ [39]. Contrary to these results, our study showed that the percentages of CD3^+^CD8^+^ T cells in spleen were increased in a dose-response manner in the four AFB-contaminated groups at the same time, The CD4^+^/CD8^+^ ratios were decreased because of the significant increase of the CD3^+^CD8^+^ T cells. CD8^+ ^T cells have been shown to play an important role in the host’s defense against malignancies [40]. CD8^+^ T cell protection mediated by its ability to speciﬁc target host cells compromised by microbial infection or oncogenic transformation [41,42]. Previous studies proved that aflatoxins present carcinogenic effects [2], and the carcinogenic potency of AFB has been observed in many species of animals, including nonhuman primates, rodents, birds and fish [2,43,44]. Therefore, the increase of CD8^+^ T cells may be related to the carcinogenic effects of AFB and autoregulation of the antitumor CD8^+^ T cell response.

## 5. Conclusions

From the results of this study, together with the above discussion, we conclude that pathological changes, increased apoptotic cells, cell cycle blockage and up-regulated CD8^+^ T cells occur in the spleen in chicks after feeding with corn contaminated with AFB_1_ and AFB_2_. The results suggested that one of the mechanisms of AFB-induced immunosuppression is splenic lesions.
